# Delayed Endovascular Stenting for Severe Vertebral Artery Stenosis with Precarious Thrombosis

**DOI:** 10.7759/cureus.1277

**Published:** 2017-05-26

**Authors:** Ali S Haider, Caleb Gottlich, Tijani Osumah, Maryam Alam, Umair Khan, Steven Vayalumkal, Dean Leonard, Richa Thakur, Kennith F Layton

**Affiliations:** 1 Texas A&M College of Medicine; 2 School of Medicine, Ross University; 3 School of Medicine, St. Georges University; 4 School of Medicine, St. George's University; 5 Department of Radiology, Baylor University Medical Center

**Keywords:** atherosclerosis, ischemic stroke, vertebrobasilar system, posterior fossa circulation

## Abstract

A significant but less recognized cause of ischemic stroke and transient ischemic attack (TIA) is atherosclerosis of the vertebrobasilar system, which accounts for 20% of ischemic strokes. Pathology of the vertebrobasilar system can present significant challenges in determining the course of treatment. Due to the complexity of the vertebrobasilar system, there is slight disagreement about how to approach patients with atherosclerotic pathology of the posterior circulation. Two such approaches are either stenting of the vertebral or basilar artery or aggressive medical management. Here, we present the case of a 63-year-old male who presented with lightheadedness, diaphoresis, two episodes of loss of consciousness, and the abrupt onset of unilateral right-sided paresis. A computed tomography angiogram (CTA) of the head and neck demonstrated complex posterior circulation vertebrobasilar vascular stenosis and occlusions. There was an unstable clot located at the junction of the vertebral and basilar arteries requiring a carefully nuanced approach. The patient was started on dual antiplatelet therapy and heparin in an effort to resolve the clot. Repeat CTA after five days revealed resolution of the unstable clot; however, the distal intradural right vertebral artery remained occluded and the left vertebral artery remained stenosed. The patient was then treated with a balloon-mounted coronary stent to eliminate the stenosis, which ultimately restored normal posterior fossa flow dynamics. This case serves as a testament to the variability and complexity of vertebrobasilar arteriopathies as well as the benefit of experienced neurointerventionalists in the successful management of these cases.

## Introduction

Intracranial atherosclerotic disease was a relatively lesser known etiology of neurological pathology until the advent of neuroimaging [1]. Neuroimaging allowed for a better understanding of the cause of ischemic events, showing atherosclerosis to be culpable a majority of the time. Most strokes are found to be ischemic in nature, with the majority of those produced from carotid vessel disease. A significant but less recognized cause of ischemic stroke and transient ischemic attack (TIA) is atherosclerosis of the vertebrobasilar system, accounting for 20% of ischemic strokes [2-4]. These patients often present with neurologically vague symptoms that point to non-neurological diagnoses, partially accounting for its diagnostic disparity [4]. Of strokes that occur in the posterior circulation, approximately one-third are due to large artery occlusive lesions, often derived from the vertebral artery, and another 14% are caused by artery-to-artery embolism [5]. Due to the complexity of the vertebrobasilar system as well as the paucity of literature on the management of patients with this presentation, there is no consensus on the management of complex posterior circulation atherosclerotic disease. Two potential approaches are either stenting of the vertebral and/or basilar arteries, which has its limitations depending on the affected arterial segment, or aggressive medical management. Vertebrobasilar system pathology can present significant challenges in determining a course of treatment and is not conducive to the formation of an algorithmic approach in contrast to other anatomic locations. These cases should be addressed individually by an experienced stroke team and often need to be examined by an interventional neuroradiologist. Here, we present a patient with posterior fossa ischemic symptoms found to have complex posterior circulation vertebrobasilar vascular stenosis and occlusions. There was an unstable clot located at the junction of the vertebral and basilar arteries, requiring a carefully nuanced management approach. This case serves as a testament to the variability and complexity of vertebrobasilar arteriopathies as well as the benefit of experienced neurointerventionalists for the management of these cases. 

## Case presentation

**Figure 1:**
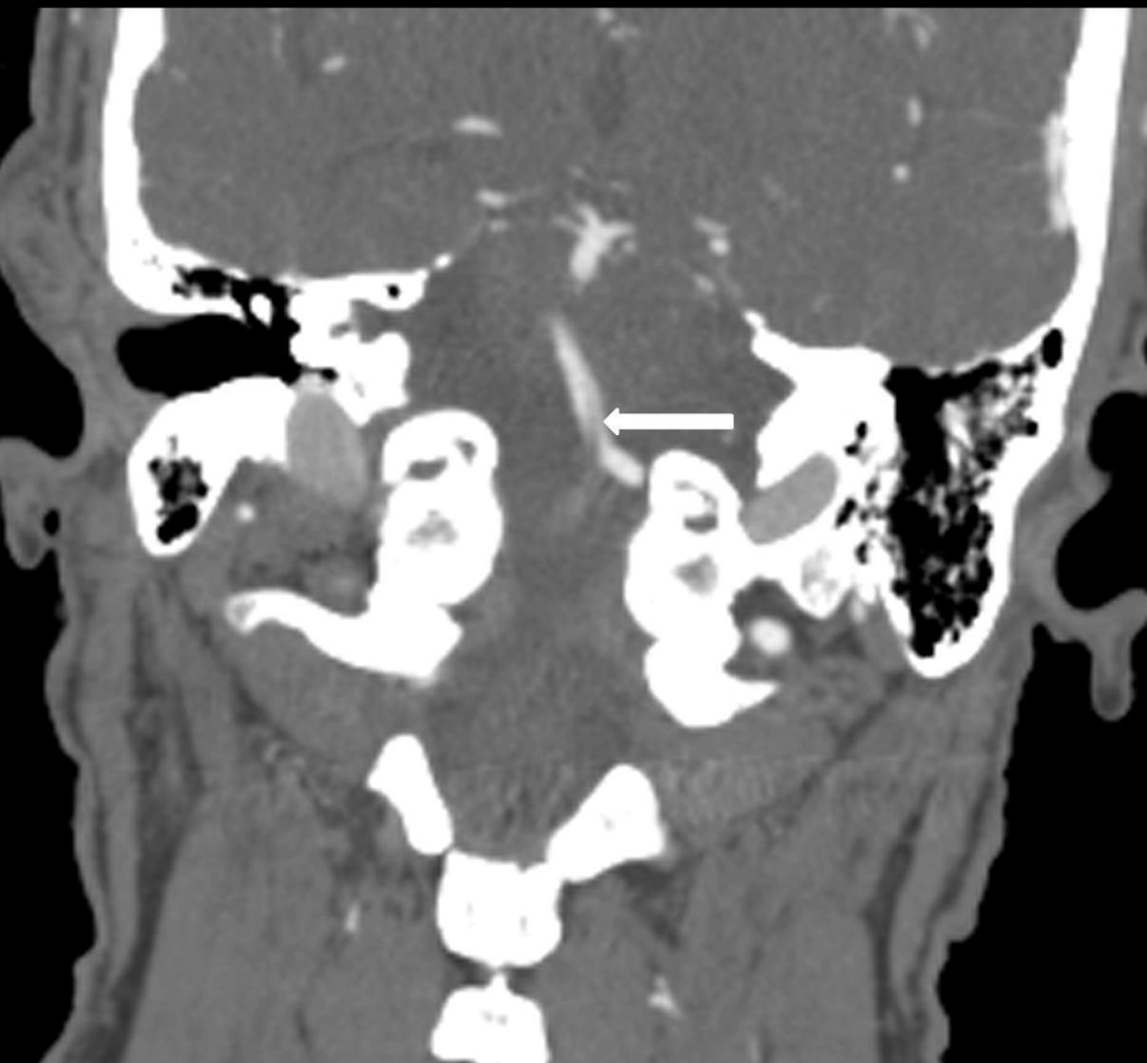
Coronal reformat computed tomography angiogram (CTA) demonstrated that the intradural right vertebral artery is completely occluded. While the right vertebral artery is completely occluded, there is also extension of partially occlusive thrombus cephalad into the proximal basilar artery at the vertebrobasilar junction (arrow).

A 63-year-old Caucasian male with two weeks of lightheadedness, diaphoresis, and two episodes of loss of consciousness presented to an outside emergency room due to an abrupt onset of unilateral paresis of the right side of the body as well as speech deficits. The patient was evaluated for stroke and deferred administration of tissue plasminogen activator (tPA) due to the length of time between onset of symptoms and presentation to the emergency department. A computed tomography angiogram (CTA) of the head and neck was ordered and demonstrated occlusion of the intradural right vertebral artery with a severe stenosis of the intradural left vertebral artery. There was a partially occlusive thrombus extending out of the occluded right vertebral artery into the proximal basilar artery (Figure 1). It was decided that the patient be transported to our high volume certified Comprehensive Stroke Center where he could receive more specialized treatment. Before transport, he regained full strength and speech which seemed to be largely dependent on blood pressure.

**Figure 2:**
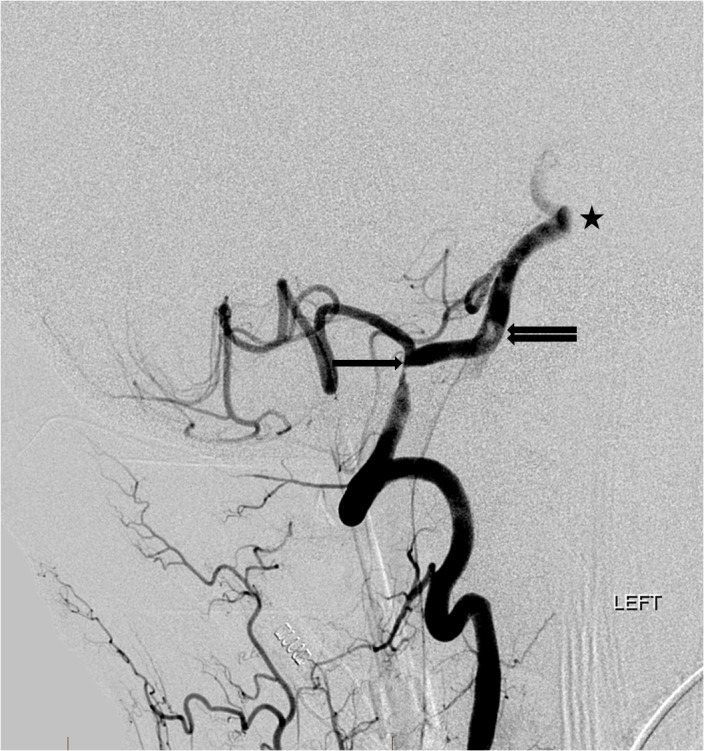
Left lateral vertebral artery digital subtraction angiogram (DSA) shows a severe stenosis of the intradural left vertebral artery (single arrow). There is also a filling defect in the proximal basilar artery (double arrow) and a congenital corkscrew shape to the mid-basilar artery (asterisk).

Upon arrival to our facility, his only complaint was paresthesia of the right foot and blurred vision as well as exacerbation of symptomology with changes in body position. A cerebral digital subtraction angiogram (DSA) revealed that the intracranial segment of the right vertebral artery was recently occluded and likely related to an underlying severe atherosclerotic stenosis near the dural ring. There was also a markedly severe stenosis of the contralateral intradural left vertebral artery (Figure 2). DSA also demonstrated the presence of an unstable, partially occlusive clot at the vertebrobasilar junction and a congenital corkscrew configuration of the mid-basilar artery distal to the clot. Due to the precarious nature of the clot in both stability and location as well as the aberrant configuration of the basilar artery prohibiting thrombectomy options, the decision was made to provide dual antiplatelet therapy and heparin for several days in an effort to resolve the unstable clot and allow for development of more favorable conditions in which stenting of the left vertebral artery lesion could occur. Care was taken to maintain the patient at a slightly elevated blood pressure in order to maintain flow through the collateral circulation and not exacerbate any further ischemic damage.

**Figure 3:**
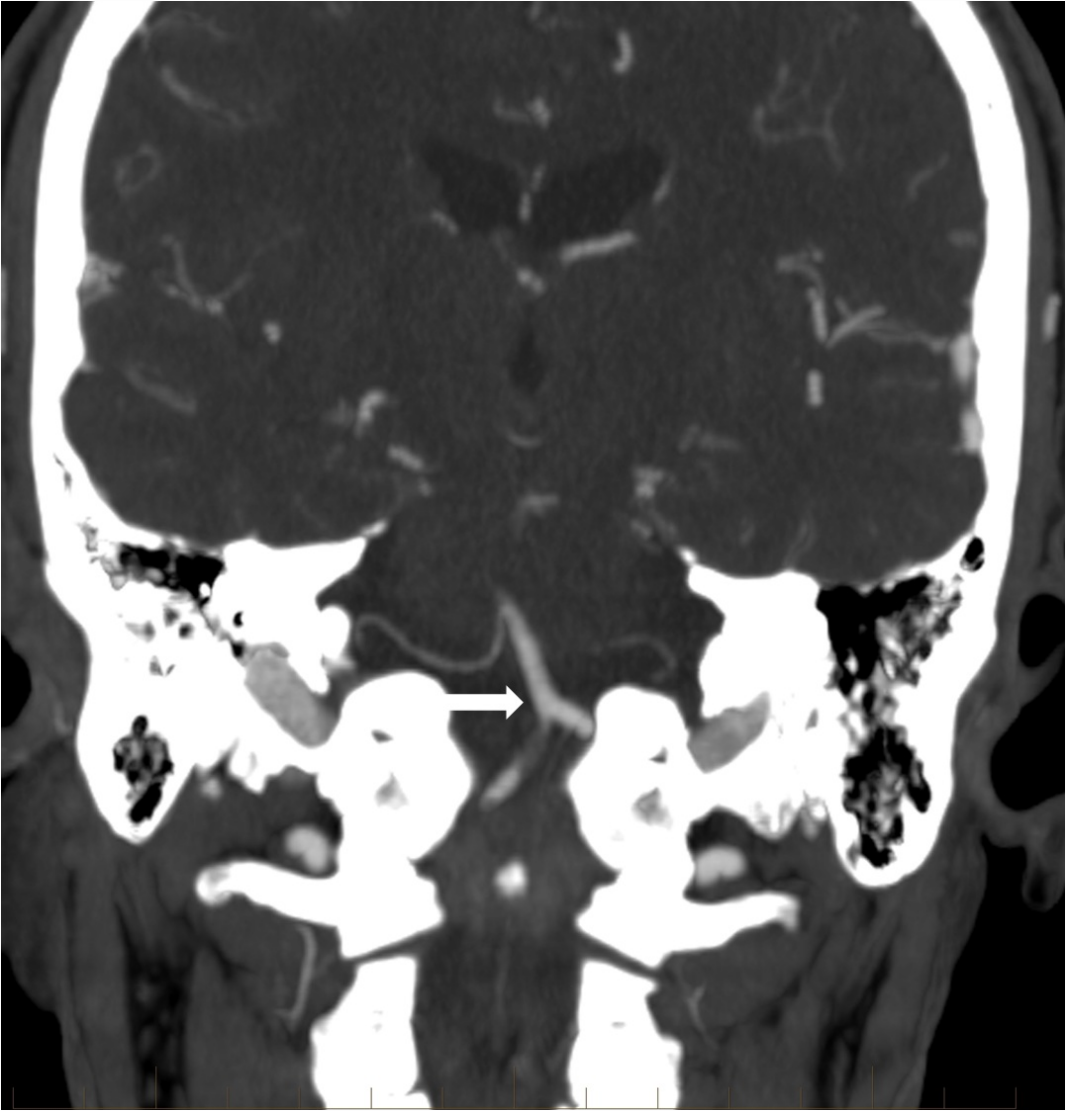
Repeat computed tomography (CT) angiogram after oral antiplatelet therapy and intravenous heparin reveals resolution of the partially occlusive thrombus in the proximal basilar artery (arrow).

**Figure 4:**
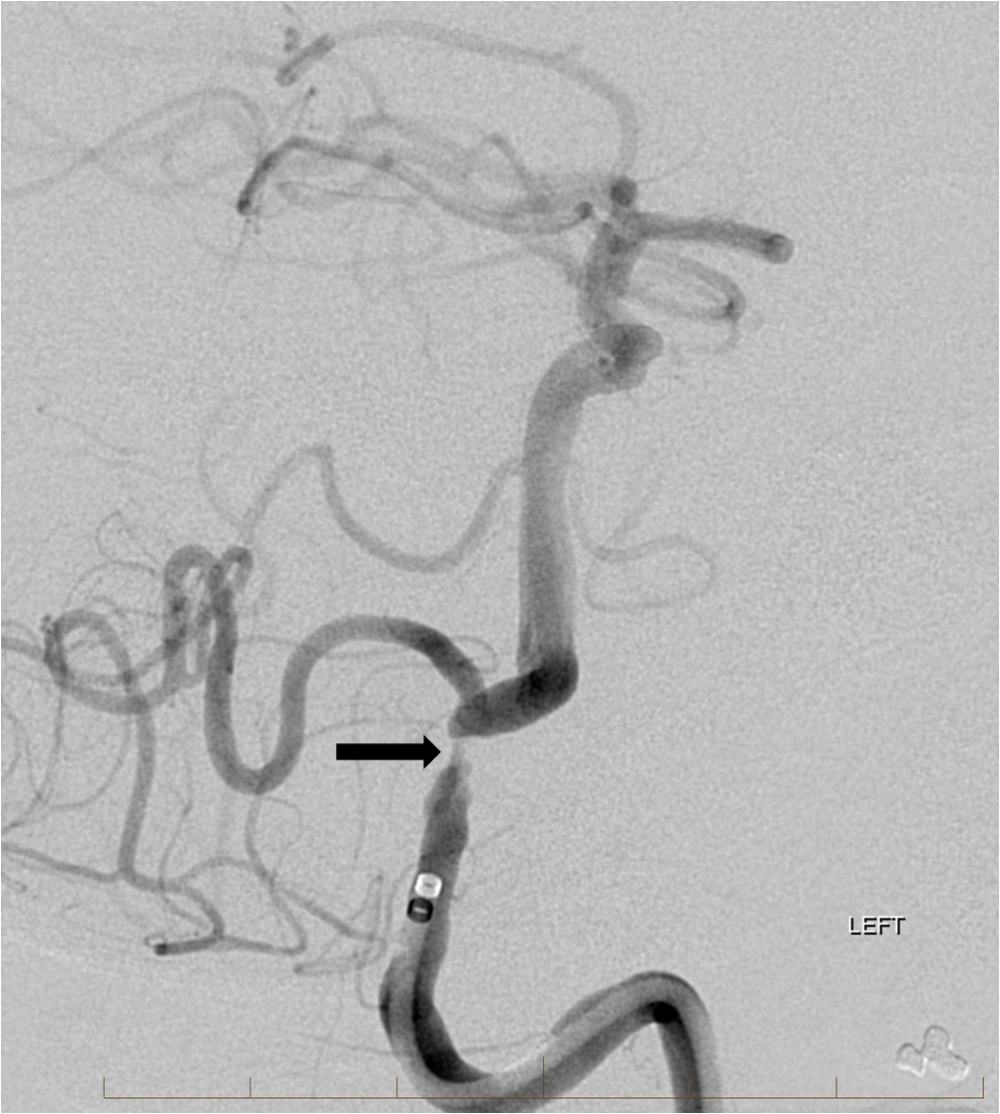
Oblique digital subtraction angiogram (DSA) after left vertebral artery injection confirms resolution of the filling defect in the proximal basilar artery and markedly severe stenosis of the left vertebral artery just proximal to the posterior inferior cerebellar artery (arrow).

**Figure 5:**
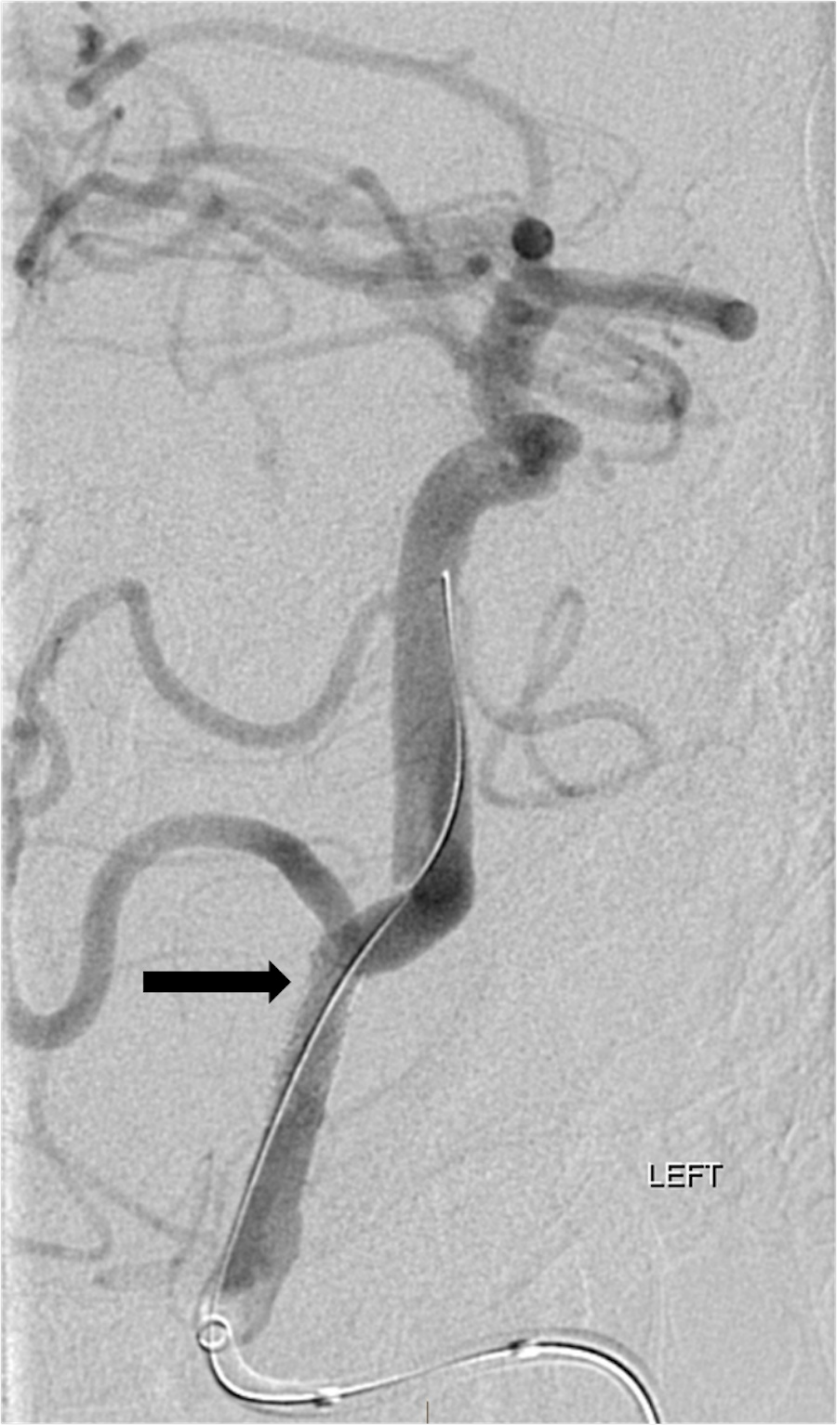
Resolution of the stenosis in left vertebral artery post stenting (arrow).

The care team developed an emergency plan for potential neurological decompensation requiring emergent transport to the interventional neuroradiology suite. Fortunately, the patient remained stable and without additional neurological deficits. Approximately five days after transfer to our facility, the patient underwent repeat CTA which revealed resolution of unstable thrombus at the vertebrobasilar junction (Figure 3). The distal intradural right vertebral artery remained occluded. The following day, the patient was brought to the interventional neuroradiology department and placed under general anesthesia with careful attention given to adequate blood pressure during induction. DSA confirmed the absence of unstable clot in the proximal basilar artery and redemonstrated the severe left vertebral artery stenosis (Figure 4). Placement of a balloon-mounted coronary stent across the severe stenosis eliminated any narrowing of the left vertebral artery (Figure 5). There was a return of normal posterior fossa flow dynamics, and the patient made a full clinical recovery.

## Discussion

There is still disagreement about how to approach patients with atherosclerotic pathology of the posterior circulation, namely, the vertebrobasilar system [5-7]. There is a high risk of stroke recurrence within this system (8.5 - 22% per year), even after medical intervention [8-9]. One potentially useful metric, analyzed in the Vertebrobasilar Flow Evaluation and Risk of Transient Ischemic Attack and Stroke (VERiTAS) Study, was that of using the flow rate through an affected vessel as a predictor for stroke likelihood [9]. This study found that low flow had a much higher likelihood of one-year stroke incidence than a normal flow vessel. This suggests that angioplasty and/or stenting could provide risk reduction of additional strokes in these specific cases.  

The vascular and clinical complexity, as well as the precisely planned measures taken to circumvent further harm to the patient, make this a noteworthy case. It also demonstrates the merit of including vertebrobasilar system pathology in the differential when appropriate for high-risk patients, as well as defending the assertions that an experienced cerebrovascular team should be involved in these cases. This case is unusual because the patient harbored stenosis of the bilateral intradural vertebral arteries with no stenosis of the much more commonly stenotic V1 or V2 segments [2, 10]. It was a calculated risk choosing to administer dual platelet and anticoagulant therapy and to wait rather than conducting the procedure immediately. The decision was made with the help of an experienced neurointerventionalist. As the thrombus was considered unstable and located at the junction of the vertebral and basilar arteries, there was concern that stenting of the severely stenosed left vertebral artery could dislodge the thrombus and cause a secondary, potentially fatal, basilar thromboembolic event. This was considered especially difficult in the presence of a congenital basilar artery corkscrew configuration which would preclude subsequent distal thrombectomy. The success of this case was contingent on the experienced counsel of the neurointerventionalist and precise communication between the healthcare team. 

## Conclusions

Ischemic strokes of the posterior circulation are less frequently encountered than their anterior circulation counterparts and often produce more ambiguous symptomology on clinical presentation. They can also be more difficult to visualize on imaging due to anatomical constraints, such as tortuosity or passage through bony structures. Due to the high mortality rate of a vertebrobasilar stroke and the devastating repercussions of an unaddressed occlusion, as well as the often ambiguous initial presentation, it is important to increase the awareness of vertebrobasilar pathology and include it in the differential diagnoses for high-risk patients. Involvement of specialists who are familiar with treating intracranial and extracranial cerebrovascular disease as well as those who are capable of accurately interpreting imaging procedures to visualize the posterior circulation is critical. A unique approach should be taken with every case of vertebrobasilar stenosis and occlusion, and consultation with an experienced neurointerventionalist at a Comprehensive Stroke Center should be considered. 

## References

[1] Wong LK (2006). Global burden of intracranial atherosclerosis. Int J Stroke.

[2] Jenkins JS, Stewart M (2017). Endovascular treatment of vertebral artery stenosis. Prog Cardiovasc Dis.

[3] Kotan D, Sayan S, Acar BA, Polat P (2013). Bilateral vertebral artery stenosis present with vertigo. BMJ Case Rep.

[4] Schoen JC, Boysen MM, Warren CR (2011). Vertebrobasilar artery occlusion. West J Emerg Med.

[5] Caplan LR, Wityk RJ, Pazdera L (2005). New England Medical Center Posterior Circulation Stroke Registry II. Vascular lesions. J Clin Neurol.

[6] Chimowitz MI, Lynn MJ, Derdeyn CP (2011). Stenting versus aggressive medical therapy for intracranial arterial stenosis. N Engl J Med.

[7] Wang ZL, Gao BL, Li TX (2015). Symptomatic intracranial vertebral artery atherosclerotic stenosis (≥70%) with concurrent contralateral vertebral atherosclerotic diseases in 88 patients treated with the intracranial stenting. Eur J Radiol.

[8] Liu L, Zhao X, Mo D (2016). Stenting for symptomatic intracranial vertebrobasilar artery stenosis: 30-day results in a high-volume stroke center. Clin Neurol Neurosurg.

[9] McMaster ML, Kristinsson SY, Turesson I (2009). Novel aspects pertaining to the relationship of Waldenstrom's macroglobulinemia, IgM monoclonal gammopathy of undetermined significance, polyclonal gammopathy, and hypoglobulinemia. Clin Lymphoma Myeloma.

[10] Gorelick PB, Wong KS, Bae HJ, Pandey DK (2008). Large artery intracranial occlusive disease: a large worldwide burden but a relatively neglected frontier. Stroke.

